# The inhibition of human lung fibroblast proliferation and differentiation by Gs-coupled receptors is not predicted by the magnitude of cAMP response

**DOI:** 10.1186/s12931-018-0759-2

**Published:** 2018-04-07

**Authors:** Maxine J. Roberts, Rebecca E. Broome, Toby C. Kent, Steven J. Charlton, Elizabeth M. Rosethorne

**Affiliations:** 1 School of Life Sciences, University of Nottingham, Nottingham, UK; 2 Novartis Institutes for Biomedical Research, Horsham, UK

**Keywords:** cAMP, Idiopathic pulmonary fibrosis, Human lung fibroblasts, G protein-coupled receptors, Drug discovery

## Abstract

**Background:**

Idiopathic pulmonary fibrosis (IPF) is a chronic and progressive fibrotic lung disease for which there is no cure. Current therapeutics are only able to slow disease progression, therefore there is a need to explore alternative, novel treatment options. There is increasing evidence that the 3′, 5′ cyclic adenosine monophosphate (cAMP) pathway is an important modulator in the development of fibrosis, with increasing levels of cAMP able to inhibit cellular processes associated with IPF. In this study we investigate the expression of G_s_-coupled G protein-coupled receptors (GPCR) on human lung fibroblasts (HLF), and explore which can increase cAMP levels, and are most efficacious at inhibiting proliferation and differentiation.

**Methods:**

Using TaqMan arrays we determined that fibroblasts express a range of G_s_-coupled GPCR. The function of selected agonists at expressed receptors was then tested in a cAMP assay, and for their ability to inhibit fibroblast proliferation and differentiation.

**Results:**

Expression analysis of GPCR showed that the prostacyclin, prostaglandin E_2_ (PGE_2_) receptor 2 and 4, melanocortin-1, β_2_ adrenoceptor, adenosine 2B, dopamine-1, and adenosine 2A receptors were expressed in HLF. Measuring cAMP accumulation in the presence of selected G_s_-coupled receptor ligands as well as an adenylyl cyclase activator and inhibitors of phosphodiesterase showed formoterol, PGE_2_, treprostinil and forskolin elicited maximal cAMP responses. The agonists that fully inhibited both fibroblast proliferation and differentiation, BAY60–6583 and MRE-269, were partial agonists in the cAMP accumulation assay.

**Conclusions:**

In this study we identified a number of ligands that act at a range of GPCR that increase cAMP and inhibit fibroblast proliferation and differentiation, suggesting that they may provide novel targets to develop new IPF treatments. From these results it appears that although the cAMP response is important in driving the anti-fibrotic effects we have observed, the magnitude of the acute cAMP response is not a good predictor of the extent of the inhibitory effect. This highlights the importance of monitoring the kinetics and localisation of intracellular signals, as well as multiple pathways when profiling novel compounds, as population second messenger assays may not always predict phenotypic outcomes.

**Electronic supplementary material:**

The online version of this article (10.1186/s12931-018-0759-2) contains supplementary material, which is available to authorized users.

## Background

Idiopathic pulmonary fibrosis (IPF) is an age-related, chronic and progressive fibrotic lung disease, with an annual incidence in the UK of 7.44 per 100,000 people, and a median survival of approximately 3 years from diagnosis [1]. The current hypothesis is that IPF is a consequence of aberrant wound healing in response to repetitive lung injury, resulting in fibrosis rather than repair. The dysregulation of the wound healing response is thought to cause the development of fibrosis through excess fibroblast proliferation, fibroblast to myofibroblast transdifferentiation (FMT), and extracellular matrix (ECM) deposition. In the IPF lung, aggregates of proliferating fibroblasts and myofibroblasts, termed “fibroblast foci”, are a key histopathological finding, which indicate that fibrosis is actively ongoing [2]. An increased accumulation of ECM and scarring in the lung, causes lung stiffness and compromises its ability to facilitate normal gas exchange [3].

A key therapeutic goal in the treatment of IPF is to reduce tissue fibrosis, which may be achieved by enhancing signals that counteract fibrotic processes, and/or inhibit signals and mediators that promote fibrosis. There are at present two drugs that are now approved for use in the treatment of IPF; nintedanib and pirfenidone. Nintedanib inhibits multiple receptor tyrosine kinases (RTK) [4] and pirfenidone has broad anti-inflammatory/anti-transforming growth factor β (TGFβ) activity [5, 6]. Although both nintedanib and pirfenidone have been shown to be efficacious in slowing the progression of disease [7–10], their non-specific action leads to severe adverse effects such as diarrhoea, dyspnoea, vomiting and weight loss, that are dose limiting thereby restricting their use to the more severe patients. Research to identify alternative pathways in IPF treatment is essential to enable the development of safer, more efficacious and better tolerated drugs.

As RTK predominantly signal through the Ras/mitogen-activated protein kinase (MAPK) signalling cascade, it is likely that this pathway is responsible for many of the pro-fibrotic responses observed in IPF [11]. There is increasing evidence that the cyclic adenosine monophosphate (cAMP) pathway can negatively impact MAPK signalling, via inhibition of extracellular signal–regulated kinase (ERK) phosphorylation and activation in certain cell types [12, 13]. This suggests that activation of the cAMP pathway could provide novel targets to treat IPF. In support of this, a number of studies have shown that cAMP can negatively regulate fibroblast function, as agonists that bind to G_s_-coupled G protein-coupled receptors (GPCR) thus activating adenylyl cyclase (AC) and increasing levels of cAMP, inhibit lung fibroblast migration, proliferation and differentiation. For example, prostaglandin E_2_ (PGE_2_) acting at PGE_2_ receptor 2 (EP_2_) and 4 (EP_4_) and iloprost acting at the prostacyclin (IP) receptor, were shown to have an inhibitory effect on proliferation and differentiation of lung fibroblasts, via cAMP accumulation and protein kinase A (PKA) activation [14–16]. Additionally, increasing levels of cAMP through direct activation of AC by forskolin inhibited the proliferation and differentiation of human embryonic fibroblasts [17]. Furthermore, inhibition of phosphodiesterases (PDE), which catalyse cAMP degradation, by roflumilast or cilomilast was shown to reduce pulmonary fibrosis in the bleomycin-induced mouse fibrosis model [18, 19].

The present study was performed in order to evaluate the anti-proliferative capacity of a range of G_s_-coupled GPCR in order to identify potential new targets to treat IPF. We have explored the ability of G_s_-coupled GPCR present in HLF to increase global cAMP using high-throughput compatible, homogenous assay kits. We have further explored how the observed increases in cAMP accumulation translate into an ability to inhibit fibroblast proliferation and differentiation, in order to determine whether any of the selected GPCR would be suitable for exploring further as novel targets to treat IPF.

## Methods

### Materials

Foetal bovine serum (FBS), trypsin-EDTA, Hoechst 33,342, RNAqueous kit, TURBO DNase and High Capacity RNA-to-cDNA kit were purchased from Life Technologies (Paisley, UK). CGS-21680, BMY-45778, BAY60–6583, iloprost, and NECA were purchased from Tocris (Abingdon, UK). Beraprost was bought from Cayman Chemical Company (Michigan, USA). PDGF-BB was purchased from R&D Systems (Minneapolis, USA). MRE-269 (CAS:475085–57-5) and AGN-205204 (CAS:802906–77-0) were synthesized in house at Novartis (Horsham, UK). Phospho-p44/42 ERK1/2 (Thr202/Tyr204) (D13.14.4E) XP® Rabbit mAb primary antibody was purchased from Cell Signalling Technology (Leiden, The Netherlands). CF™647-conjugated Affini-pure goat anti-rabbit IgG and CF™647-conjugated Affini-pure goat anti-mouse IgG were purchased from Biotium, Inc., (Fremont, California). Pierce™ LDH Cytotoxicity Assay Kit and CellEvent™ Caspase-3/7 Red Detection Reagent were purchased from ThermoFisher (Hemel Hempstead, UK). Staurosporine was purchased from VWR International Ltd. (Lutterworth, UK). 96-well white ViewPlates, AlphaScreen cAMP and DELFIA proliferations assay kits were purchased from Perkin Elmer Life Sciences (Massachusetts, USA), 96-well black ViewPlates were purchased from Corning (New York, USA), and 384-well black, μ-clear Viewplates were purchased from Greiner bio-one (Stonehouse, UK). HitHunter™ cAMP assay kits were purchased from DiscoverX (Birmingham, UK). All other reagents were purchased from Sigma Aldrich (Poole, UK).

### Cell culture

All studies were performed on normal human lung fibroblasts (C-12361), which were purchased from Promocell (Heidelberg, Germany). HLF were maintained in DMEM supplemented with 4.5 g L^− 1^ D-glucose, L-glutamine, pyruvate, FBS (10% *v*/v), and 25 mM HEPES at 37°C, 5% CO_2_, in a humidified atmosphere. For experiments cells were harvested using trypsin/EDTA and seeded in their sub-culture medium. Two HLF donors were used in the functional studies: HLF d91019001.2 and HLF 9017402.1, both from healthy/non-smoker Caucasian donors.

### Receptor expression levels

Expression of GPCR was determined in HLF using high density, 384-well GPCR TaqMan arrays as previously described [20]. RNA from 4 fibroblast donors (all from healthy/non-smoker Caucasian donors) was isolated using the RNAqueous kit and treated with TURBO DNase to remove genomic DNA. Using a High Capacity RNA-to-cDNA kit according to manufacturer’s instructions to synthesize cDNA. The GPCR array contained validated primer/probe sets for 367 GPCR. 100 ng of cDNA was loaded per port and the array was run on an Applied Biosystems 7900HT fast real-time PCR instrument according to the manufacturer’s instruction. 13 housekeeping genes were included as controls in the arrays for the purpose of quality control and data normalisation: (ACTB, β-actin; B2M, β-2-microglobin; GAPDH, glyceraldehyde-3-phosphate dehydrogenase; GUSB, β-Glucuronidase; HMBS, hydroxymethylbilane synthase; HPRT1, hypoxanthine phosphoribosyltransferase 1; IPO8, importin 8; PGK1, phosphoglycerate kinase 1; POLR2A, RNA polymerase II subunit A; PPIA, peptidylprolyl isomerase A; RPLPO, ribosomal protein lateral stalk subunit P0; TBP, TATA-Box binding protein; TFRC, Transferrin Receptor). Data was analyzed using RQ manager version 1.2 and DataAssist version 2 (Applied Biosystems) with a Cycle Threshold (C_T_) set at 0.2 for all samples.

### ERK phosphorylation

HLF were seeded overnight at 6000 cells/well in 96-well black clear bottom plates then starved for 24 h in the growth medium devoid of all additives. To assess ERK phosphorylation (pERK), cells were incubated in growth medium devoid of all additives (+ 0.1% HSA, sterile filtered) for 30 min with a range of concentrations of forskolin. After this time, cells were stimulated with an EC_80_ concentration of PDGF (3 ng/mL) for 10 min, at 37°C, 5% CO_2_. After stimulation, cells were fixed in 4% paraformaldehyde, washed 3× in PBS (with Ca^2+^/Mg^2+^), and incubated with permeabilising blocking buffer (dPBS (with Ca^2+^/Mg^2+^), 10% FBS (*v*/v), 0.1% Triton X-100 (v/v)) for 1 h at 37°C. Wells were then washed 3× in wash buffer (Tris Buffered Saline (TBS), 0.05% (v/v) Tween-20) and incubated with rabbit anti-pERK1/2 antibody (1:1000 dilution in blocking buffer overnight at 4°C. Following 3× wash in wash buffer, wells were incubated with blocking buffer containing Hoechst to stain for nuclear DNA (2 μM), and FITC-conjugated Affini-pure goat anti-rabbit IgG (1:1000 dilution in blocking buffer) for 30 min at 37°C. Fluorescence was then quantified on the InCell2000, using DAPI settings to visualize nuclei (0.1 s exp) and FITC settings for pERK (2 s exp).

To quantify levels of pERK fluorescence, a standard Multi-Target Analysis algorithm was used in the INCell Analyzer Workstation (v3.7.1). To account for the inter-assay variation in levels of ERK phosphorylation produced in each experiment, data were normalized to the EC_80_ PDGF response.

### cAMP accumulation

HLF were grown to confluency in 96-well white ViewPlates, before incubation with a range of agonist concentrations, or vehicle, for 2 h at room temperature in HBSS with 5 mM HEPES, 0.1% *w*/*v* HSA, and 5 μM rolipram. Rolipram was included in these assays to inhibit PDE activity, which resulted in increased maximal responses of cAMP accumulation, without any effect on EC_50_ values generated (data not shown). Rolipram was excluded when measuring cAMP levels in response to PDE inhibitors. cAMP levels were measured using either HitHunter cAMP assay or AlphaScreen competition assay following manufacturer protocol, and were assessed on either a BMG LABTEK ClarioStar or a Packard EnVision plate reader. cAMP concentrations in each well were determined using a standard curve. To account for the inter-assay variation in levels of cAMP produced in each experiment, data were normalized to the maximal forskolin response.

### Proliferation

Proliferation of HLF was measured by incorporation of bromodeoxyuridine (BrdU). HLF were seeded overnight at 4000 cells/well in 96-well black ViewPlates, before being starved for 24 h in culture medium devoid of FBS. Proliferation was measured after incubation with a range of concentrations of FBS or PDGF, in DMEM supplemented with 0.1% *w*/*v* HSA, for 24 h.

For anti-remodelling assays using BrdU incorporation, cells were incubated for 24 h with a range of concentrations of test compounds in the presence of near maximal concentrations of FBS or PDGF in DMEM supplemented with 0.1% w/v HSA. Proliferation was assessed using the DELFIA BrdU incorporation assay kit following manufacturer protocol. Fluorescence was measured using a Packard EnVision plate reader. To account for the inter-assay variation in levels of proliferation in each experiment, data were normalized to the PDGF or FBS response.

### Fibroblast to myofibroblast transdifferentiation

To assess the differentiation of fibroblasts to the myofibroblast phenotype, immunofluorescence was used to monitor increases in α smooth muscle actin (αSMA). HLF were seeded overnight at 1000 cells/well in 384-well black clear bottom plates, before being starved for 24 h growth medium devoid of FBS.

For anti-remodelling assays using FMT, cells were incubated for 48 h with a range of concentrations of test compounds and incubated for 48 h, at 37°C, 5% CO_2_ in the presence of a near maximal concentration of TGFβ (0.3 ng/mL), to promote differentiation in in DMEM supplemented with 0.1% *w*/*v* HSA. Following stimulation, cells were fixed in 4% paraformaldehyde, washed 3× in PBS (with Ca^2+^/Mg^2+^), and incubated with permeabilising blocking buffer (dPBS (with Ca^2+^/Mg^2+^), 10% FBS (*v*/v), 0.1% Triton X-100 (v/v)) for 1 h at 37°C. Wells were then washed 3× in wash buffer (Tris Buffered Saline (TBS), 0.05% (v/v) Tween-20) and incubated with mouse anti-α SMA (1:1000 dilution in permeabilising blocking buffer) for 1 h at room temperature, with gentle shaking. Following 3× wash in wash buffer, wells were incubated with blocking buffer containing Hoechst to stain for nuclear DNA (2 μM), and FITC-conjugated Affini-pure goat anti-rabbit IgG (1:1000 dilution in blocking buffer) for 30 min at 37°C. Fluorescence was then quantified on a widefield ImageXpress Micro microscope, with a Plan Fluor 20X objective, DAPI filter cube (excitation 400–418 nm, emission 435–470 nm), 50 ms exposure time for nuclei, and FITC filter cube (excitation 467–498 nm, emission 513–556 nm), 200 ms exposure time for αSMA.

To quantify increases in αSMA fluorescence, a standard Multi-Wavelength Cell Scoring algorithm was used in MetaXpress 5.3 software (Molecular Devices, California, USA). To account for the inter-assay variation in levels of αSMA expression produced in each experiment, data were normalized to the response to TGFβ.

### Data analysis

Data were analysed using GraphPad Prism (Version 7.00) and results expressed as mean ± standard error of mean (SEM), unless otherwise stated. Concentration-response data were fitted using a four-parameter logistic equation. Expression of GPCR mRNA is reported as 2 TO - ΔCT compared to the mean Cycle Threshold (CT) of the housekeeping genes and each value is the mean ± SD of 4 biological replicates. The Pearson correlation coefficient (r) was determined using the standard correlation function in GraphPad prism, followed by a two-tailed T test to determine significance.

## Results

### Role of cAMP in the inhibition of ERK phosphorylation, and ERK phosphorylation in proliferation

**Fig. 1:**
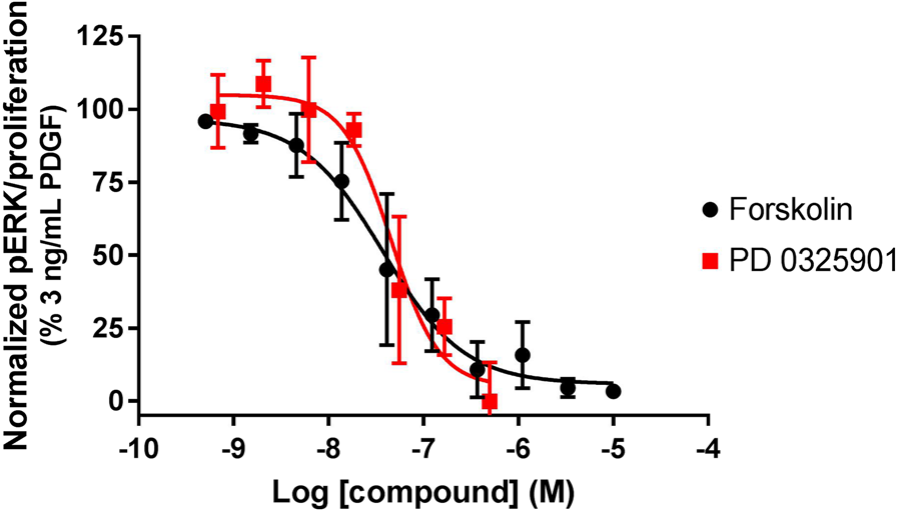
Inhibition of ERK1/2 phosphorylation and proliferation. Concentration effect curves for forskolin-mediated inhibition of ERK phosphorylation, and PD0325901-mediated inhibition of proliferation in HLF in the presence of 3 ng/mL PDGF. For each individual experiment, data were normalised to the maximum amount of ERK phosphorylation or proliferation after addition of 3 ng/mL PDGF, and are expressed as means ± SEM for 4 independent experiments

We first aimed to determine whether cAMP accumulation leads to inhibition of the pERK signal cascade in HLF, which may in turn suggest a role for cAMP in the inhibition of proliferation. Inhibition of ERK phosphorylation by the MEK inhibitor PD0325901 blocked PDGF-mediated proliferation of HLF. In turn forskolin, which increases cAMP through direct activation of AC, inhibited PDGF-mediated ERK phosphorylation (Fig. 1). For this reason, we explored the expression of GPCR in HLF, in order to determine which receptors would have the potential to increase cAMP and hence inhibit HLF proliferation and differentiation.

### GPCR expression in HLF

We first established the expression profile of GPCR mRNA in HLF (See Additional file 1: Table S1) to select receptors to study further. All GPCR tested were characterised according to the canonical G protein pathway they are reported to couple to [21]. The G_s_-coupled receptors expressed in HLF from high expression to low expression were IP, EP_2_, melanocortin-1 (MC1), β_2_ adrenoceptor, adenosine 2B (A_2B_), EP_4_, dopamine-1, and adenosine 2A (A_2A_) (Table 1).

**Table 1:** Ligands used in these studies

Ligand	Target	Mechanism of action
Iloprost	IP = EP_1_ > EP_3_ > FP > EP_4_ > TP > EP_2_	Agonist
Treprostinil	IP > EP_2_ > EP_3_ > EP_4_ > EP_1_ > TP > FP	Agonist
MRE-269	IP	Agonist
BMY-45778	IP	Agonist
Beraprost	IP	Agonist
PGE_2_	EP_1–4_	Agonist
Misoprostol	EP_2_ > EP_3_ > EP_4_ > EP_1_	Agonist
Butaprost	EP_2_, EP_4_	Agonist
AGN-205204	EP_4_	Agonist
α-MSH	MC1, MC3, MC4, MC5	Agonist
Formoterol	β_2_	Agonist
Indacaterol	β_2_	Agonist
Salbutamol	β_2_	Agonist
Salmeterol	β_2_	Agonist
NECA	A_3_, A_1_, A_2A_, A_2B_	Agonist
CGS-21680	A_2A_	Agonist
BAY60–6583	A_2B_	Agonist
Dopamine	D_1_ = D_5_ > D_3_ > D_2_ > D_4_	Agonist
Forskolin	AC 1–8	Activator
IBMX	PDE_4_, PDE_3_, PDE_1_, PDE_5_, PDE_2_	Inhibitor
Rolipram	PDE_4_	Inhibitor

Ligands used in this study, their molecular targets and mechanism of action

The IP receptor was expressed at the highest level, with 2TO-ΔC_T_ of 0.053, approaching twice as much as the second highest expressed receptor EP_2_, with 2TO-ΔC_T_ of 0.029. All the other receptors expressed had 2TO-ΔC_T_ that ranged from 0.012 for the MC1 receptor to 0.002 for the A_2A_ receptor. Based on these results, a range of ligands were chosen that target these receptors (Table 2).

**Table 2:** Expression analysis of selected GPCR in normal HLF

Receptor	Expression (2 TO – ΔC_T_)
IP	0.053 ± 0.016
EP_2_	0.029 ± 0.001
MC1	0.012 ± 0.002
β_2_	0.008 ± 0.004
A_2B_	0.007 ± 0.003
EP_4_	0.004 ± 0.002
Dopamine-1	0.004 ± 0.001
A_2A_	0.002 ± 0.001

Expression analysis of endogenously expressed G_s_-coupled receptors in HLF. High density 384-well GPCR TaqMan arrays were run on normal HLF to monitor the expression of a range of G_s_-coupled receptors. Expression is reported as 2 TO - ΔC_T_ compared to the mean Cycle Threshold (C_T_) of the 13 housekeeping genes that were included as controls in the arrays, and each value is the mean ± SD of 4 biological replicates

### cAMP signalling in HLF

**Fig. 2:**
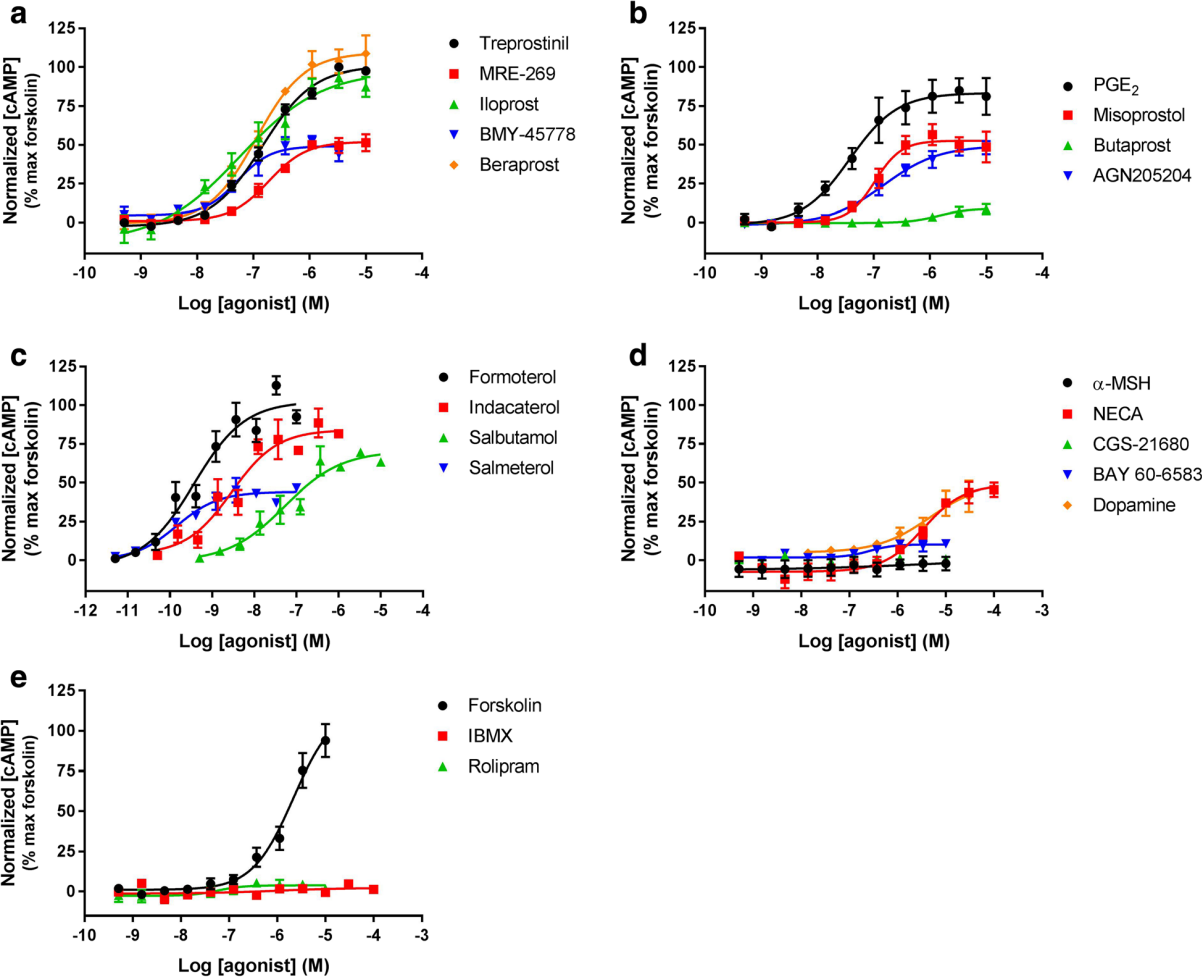
Receptor-mediated cAMP accumulation. Concentration effect curves for cAMP accumulation in HLF after treatment with a range of agonists targeting (**a**) IP receptor, (**b**) EP receptor, (**c**) β_2_ adrenoceptor, (**d**) other GPCR and (**e**) non-receptors. Data for each individual experiment were normalized to maximal cAMP accumulation observed with 10 μM forskolin, and are expressed as mean ± SEM for at least 3 independent experiments

In order to investigate the functional activity of the receptors that demonstrated expression at the mRNA level, cAMP accumulation was measured in HLF in the presence of selected G_s_-coupled GPCR agonists (Fig. 2). cAMP was monitored using commonly used, high throughput compatible, homogenous assay systems that allow quantification of cAMP in both cell media and lysates. In addition, cAMP accumulation was also investigated through direct activation of AC and inhibition of PDE, using forskolin and IBMX or rolipram, respectively. The majority of agonists tested increased cAMP levels in a concentration-dependent manner, with a range of potencies and efficacies (see Table 3). However, targeting the A_2A_ receptor with CGS21680 and the MC1 receptor with α-MSH resulted in no detectable cAMP response. Furthermore, inhibiting PDE with IBMX or rolipram resulted in no detectable cAMP accumulation. Forskolin, formoterol, treprostinil, PGE_2_ and iloprost were fully efficacious in this assay, with other ligands giving partial responses that ranged from 8.76 to 83.3% of the forskolin response.

**Table 3:** Potency and intrinsic activity of all ligands for cAMP accumulation and inhibition of proliferation assays, in HLF

Agonist	cAMP		Proliferation vs serum		Proliferation vs PDGF	
	pEC_50_	E_max_ (% max)	pEC_50_	E_max_ (% max)	pEC_50_	E_max_ (% max)
Treprostinil	6.82 ± 0.06	101 ± 0.58	6.99 ± 0.27	42.5 ± 5.36	7.53 ± 0.12	63.7 ± 6.39
MRE-269	6.75 ± 0.15	52.9 ± 4.36	NC	NC	6.50 ± 0.23	85.7 ± 7.65
Iloprost	7.11 ± 0.34	99.5 ± 7.22	NC	NC	NC	NC
BMY-45778	7.25 ± 0.09	50.0 ± 4.59	ND	ND	ND	ND
Beraprost	6.98 ± 0.08	107 ± 8.99	ND	ND	ND	ND
PGE_2_	7.34 ± 0.10	85.5 ± 8.64	7.04 ± 0.18	43.1 ± 6.14	8.60 ± 0.08	71.0 ± 3.86
Misoprostol	6.97 ± 0.06	52.4 ± 6.23	6.74 ± 0.10	34.7 ± 0.71	6.13 ± 0.04	94.1 ± 1.59
Butaprost	5.81 ± 0.16	8.76 ± 3.25	7.19 ± 0.38	29.1 ± 7.29	NC	NC
AGN-205204	6.62 ± 0.12	49.7 ± 3.84	6.91 ± 0.48	51.3 ± 8.89	7.45 ± 0.25	101 ± 3.71
Formoterol	9.38 ± 0.20	103 ± 4.60	9.58 ± 0.29	37.2 ± 2.64	9.08 ± 0.72	70.1 ± 2.96
Indacaterol	8.58 ± 0.20	83.3 ± 4.47	7.96 ± 0.35	47.9 ± 1.58	8.84 ± 0.28	90.3 ± 5.25
Salbutamol	7.36 ± 0.34	68.9 ± 1.40	7.04 ± 0.57	32.4 ± 5.84	7.98 ± 0.17	69.2 ± 5.53
Salmeterol	9.91 ± 0.16	45.8 ± 3.53	9.58 ± 0.14	37.7 ± 7.33	10.5 ± 0.15	89.2 ± 0.27
α-MSH	NC	NC	NC	NC	NC	NC
NECA	5.39 ± 0.13	47.3 ± 3.40	NC	NC	6.94 ± 0.43	37.8 ± 14.7
CGS-21680	NC	NC	NC	NC	NC	NC
BAY60–6583	6.57 ± 0.06	10.2 ± 2.89	5.30 ± 0.16	100 ± 0.01	5.30 ± 0.12	100 ± 0.01
Dopamine	5.39 ± 0.07	41.2 ± 10.0	ND	ND	4.78 ± 0.27	73.5 ± 13.5
Forskolin	NC	NC	5.28 ± 0.13	74.0 ± 6.70	5.98 ± 0.14	90.8 ± 9.12
IBMX	NC	NC	NC	NC	NC	NC
Rolipram	NC	NC	NC	NC	NC	NC

Potency and intrinsic activity values for a range of G_s_-coupled receptor agonists, PDE inhibitors, and forskolin in cAMP accumulation and BrdU inhibition assays in HLF. For cAMP accumulation intrinsic activity was calculated as a percentage of the maximal forskolin response. For BrdU inhibition intrinsic activity was calculated as a percentage of maximal proliferation observed with serum or PDGF, as indicated. Data were expressed as means ± SEM for at least 3 independent experiments

*NC* not calculated due to incomplete curve, *ND* not determined in this assay

### Proliferation

To determine if the cAMP response we observed in HLF was able to inhibit processes associated with airway remodelling, we investigated the ability of the same range of G_s_-coupled GPCR agonists, as well as PDE inhibitors and AC activators, to inhibit HLF proliferation.

We first monitored cell number over time using a nuclei count assay after treatment with a range of concentrations of FBS or PDGF for 24, 48, 72, or 96 h (Additional file 1: Figure S2). There was no detectable increase in number of HLF after 24 h treatment with either of the stimuli used. FBS was capable of stimulating proliferation in a concentration-dependent manner at 48, 72 and 96 h (Additional file 1: Figure S2A), with EC_50_ of 0.62 ± 0.08% *v*/v at 48 h, and estimated EC_50_ of 3.42 ± 0.77% v/v, and 4.97 ± 0.92% v/v, respectively. In contrast, when treated with PDGF, HLF proliferation was only observed after 72 and 96 h treatment, with EC_50_ of 3.77 ± 0.13 ng/ml and 7.26 ± 3.23 ng/ml, respectively (Additional file 1: Figure S2B). The PDGF response was partial compared to the FBS response, with maximal proliferation of 29.2 ± 15.2% of the maximal serum response observed after 96 h treatment.

HLF were then treated for 48 h with a range of concentrations of ligands in the presence of an FBS concentration (1.8% *v*/v) that relates to near maximal proliferation (as determined from Additional file 1: Figure S2A). Agonists and PDE inhibitors inhibited FBS-driven proliferation with varying levels of efficacy and potency, however, full concentration-response curves could not be established for most agonists (Additional file 1: Figure S3), and therefore accurate EC_50_ and E_max_ values could not be deduced. For this reason, we explored the use of an alternative assay to monitor fibroblast proliferation.

**Fig. 3:**
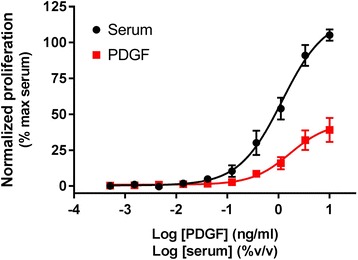
Fibroblast proliferation. Concentration-dependent increase in HLF proliferation following treatment with serum or PDGF for 24 h, assayed using BrdU incorporation. Data were normalized to maximal proliferation observed with 10% serum, and expressed as mean ± SEM for 8 independent experiments

By monitoring BrdU incorporation into newly synthesized DNA of actively proliferating cells, we demonstrated that treatment of HLF with FBS or PDGF resulted in increases in proliferation in a concentration-dependent manner after 24 h incubation (Fig. 3). Similar to the nuclei count assays, PDGF was partial in comparison to FBS, with maximal proliferation of 41.0 ± 8.62% and 112 ± 5.49% for PDGF and FBS respectively.

**Fig. 4:**
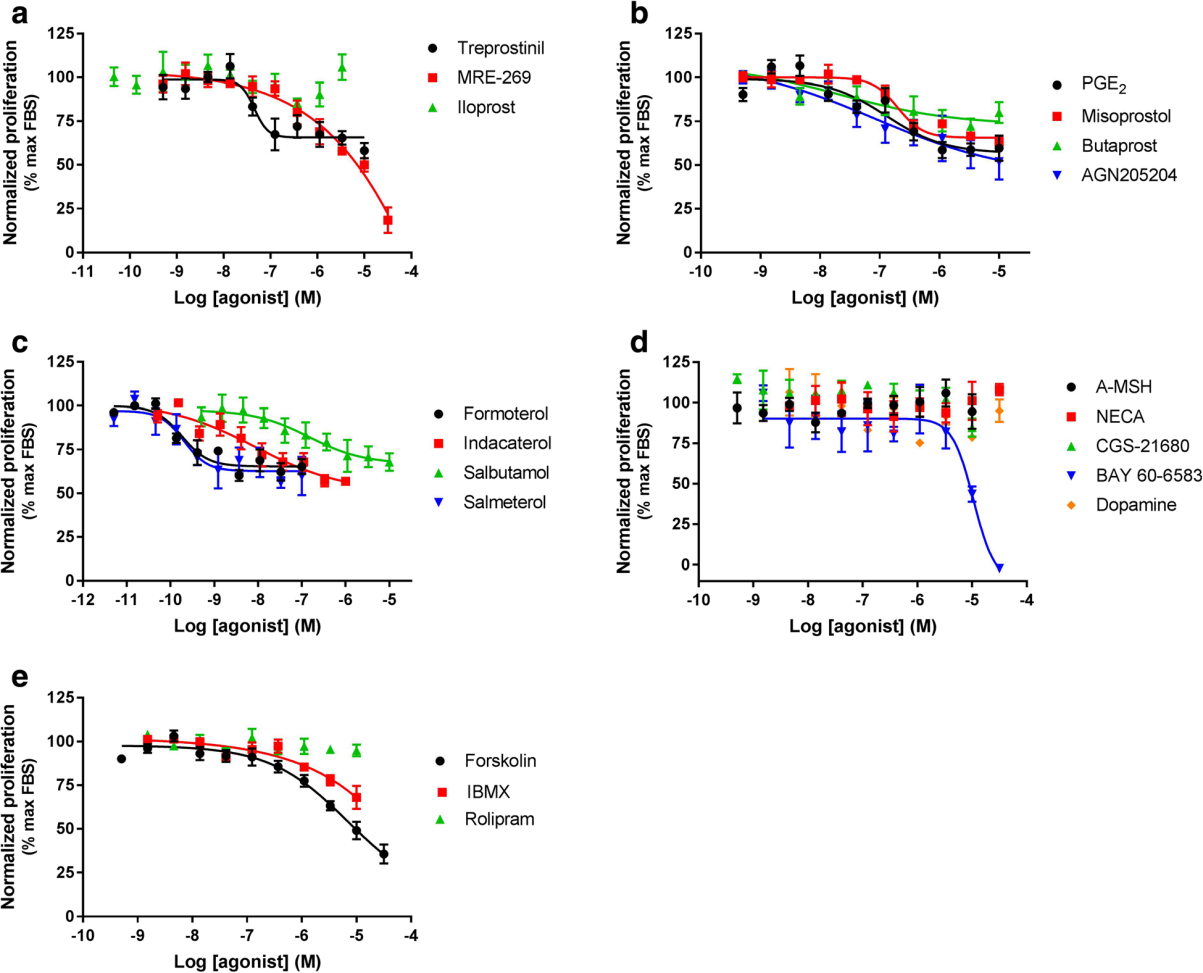
Receptor-mediated inhibition of serum-mediated proliferation. Concentration effect curves for the inhibition of proliferation were determined in HLF following exposure to a range of agonists targeting the (**a**) IP receptor, (**b**) EP receptor, (**c**) β_2_ adrenoceptor, (**d**) other GPCR and (**e**) non-receptors, in the presence of an EC_80_ concentration of serum for 24 h. For each individual experiment, data were normalised to the maximal proliferation observed with serum, and are expressed as means ± SEM for at least 3 independent experiments

**Fig. 5:**
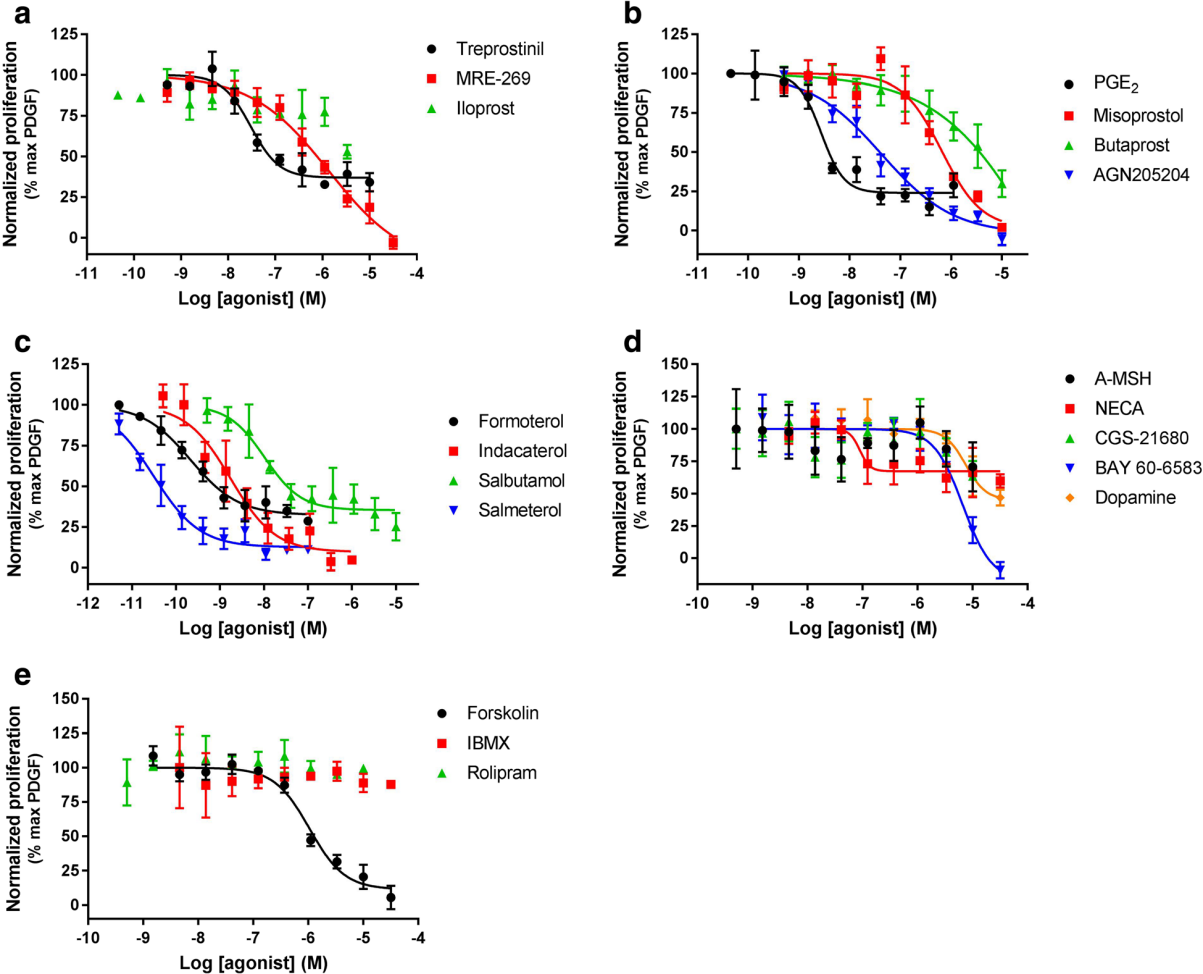
Receptor-mediated inhibition of PDGF-mediated proliferation. Concentration effect curves for the inhibition of proliferation were determined in HLF following exposure to a range of agonists targeting the (**a**) IP receptor, (**b**) EP receptor, (**c**) β_2_ adrenoceptor, (**d**) other GPCR and (**e**) non-receptors, in the presence of an EC_80_ concentration of PDGF for 24 h. For each individual experiment, data were normalised to the maximal proliferation observed with PDGF, and are expressed as means ± SEM for at least 3 independent experiments

HLF were then treated for 24 h with a range of concentrations of ligands in the presence of near maximal concentration of FBS or PDGF (as determined from Fig. 3). Targeting the IP, EP_2_, EP_4_, β_2_, A_2B_, and dopamine receptors, as well as activating AC, inhibited HLF proliferation with varying degrees of intrinsic activity and potency (Table 3; Figs. 4 and 5). When inhibiting serum-driven proliferation, ligands achieved maximal levels of inhibition ranging from 100% for BAY60–6583 to 29% for butaprost. After treatment with PDGF maximal levels of inhibition ranged from 100% for BAY60–6583 to 38% for NECA. BAY60–6583 was the only ligand to fully inhibit both FBS- and PDGF-driven proliferation, with other ligands being more efficacious at inhibiting PDGF-driven proliferation. α-MSH and rolipram were incapable of inhibiting the proliferation mediated by either stimulus.

In order to confirm that test compounds were directly inhibiting HLF proliferation rather than promoting cell death, cell viability was measured using the presence of caspase-3/7 as markers of apoptosis. None of the compounds tested caused a significant increase in expression of caspase-3/7, whereas treatment with the toxic compound staurosporine resulted in statistically significant cell apoptosis (*P* < 0.001) (See Additional file 1: Table S2).

### Fibroblast to myofibroblast transdifferentiation

IPF is driven by a number of pro-fibrotic processes, therefore we next aimed to determine if the same agonists were also capable of inhibiting another key component of fibrosis, TGFβ-induced FMT. TGFβ is a key driver of fibrosis and was able to robustly promote FMT in HLF with EC_50_ equal to 0.07 ± 0.01 ng/mL (data not shown).

**Fig. 6:**
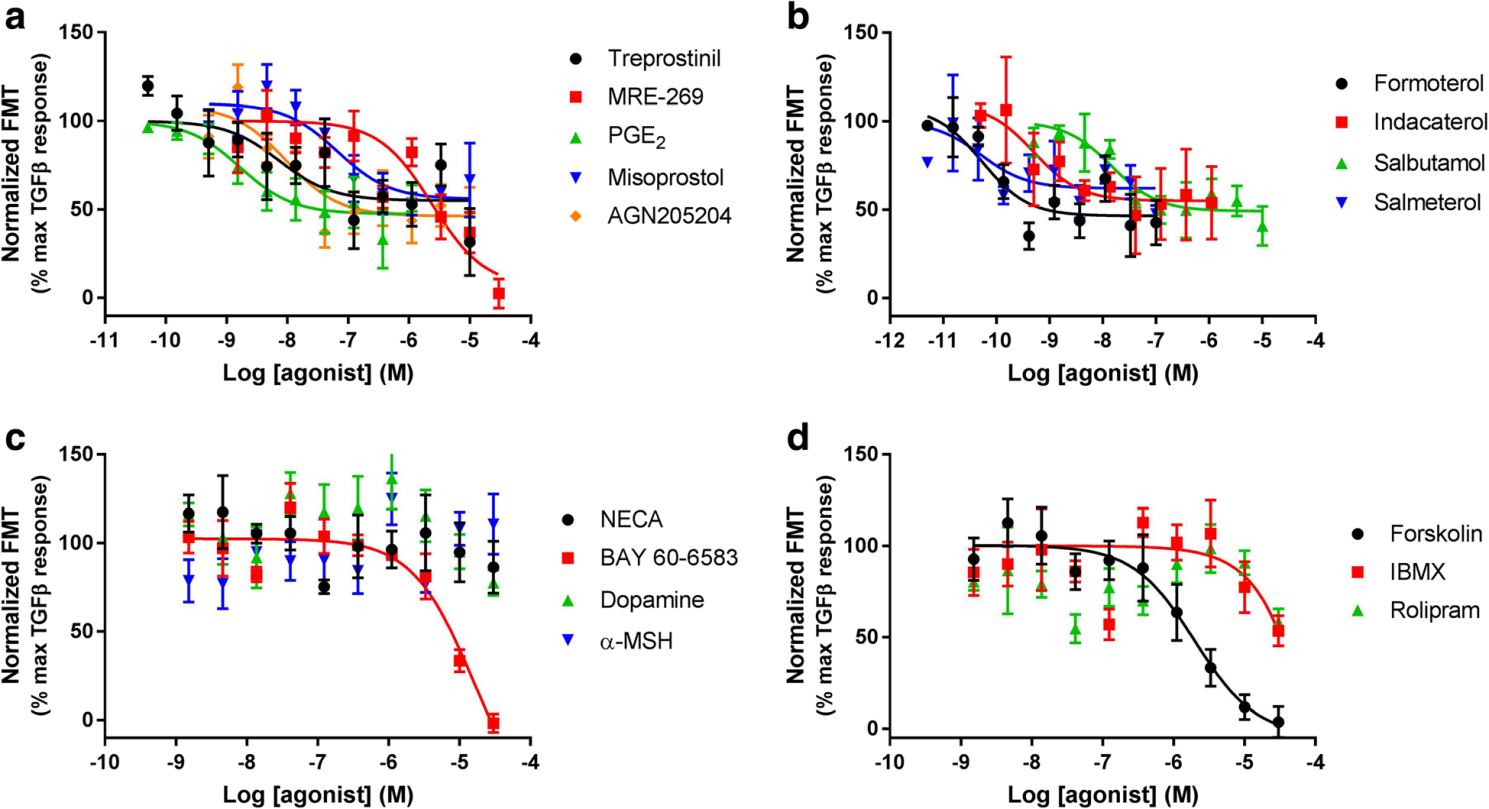
Receptor-mediated inhibition of TGFβ-mediated FMT. Concentration effect curves for the inhibition of FMT were determined in HLF following exposure to a range of agonists targeting the (**a**) IP or EP receptors, (**b**) β_2_ adrenoceptor, (**c**) other GPCR and (**d**) non-receptors, in the presence of an EC_80_ concentration of TGFβ for 48 h. For each individual experiment, data were normalised to the maximal levels of FMT observed with TGFβ, and are expressed as means ± SEM for 4 independent experiments

In order to test the ability of compounds to inhibit FMT, HLF were treated for 48 h with a range of concentrations of the selected ligands in the presence of a TGFβ concentration (0.3 ng/mL) that relates to near maximal FMT. Agonists and PDE inhibitors inhibited TGFβ-driven FMT with varying levels of efficacy and potency (Fig. 6), however, full concentration-response curves could not be established for NECA, dopamine, α-MSH, IBMX or rolipram. Of the agonists tested, MRE-269, Bay 60–6583 and forskolin were able to completely inhibit FMT. All other ligands were able to partially inhibit FMT, with maximal levels of inhibition ranging from 71% of the TGFβ response for AGN205204 to 51% of the TGFβ response for salmeterol.

### Correlation between cAMP and the inhibition of proliferation and differentiation

**Fig. 7:**
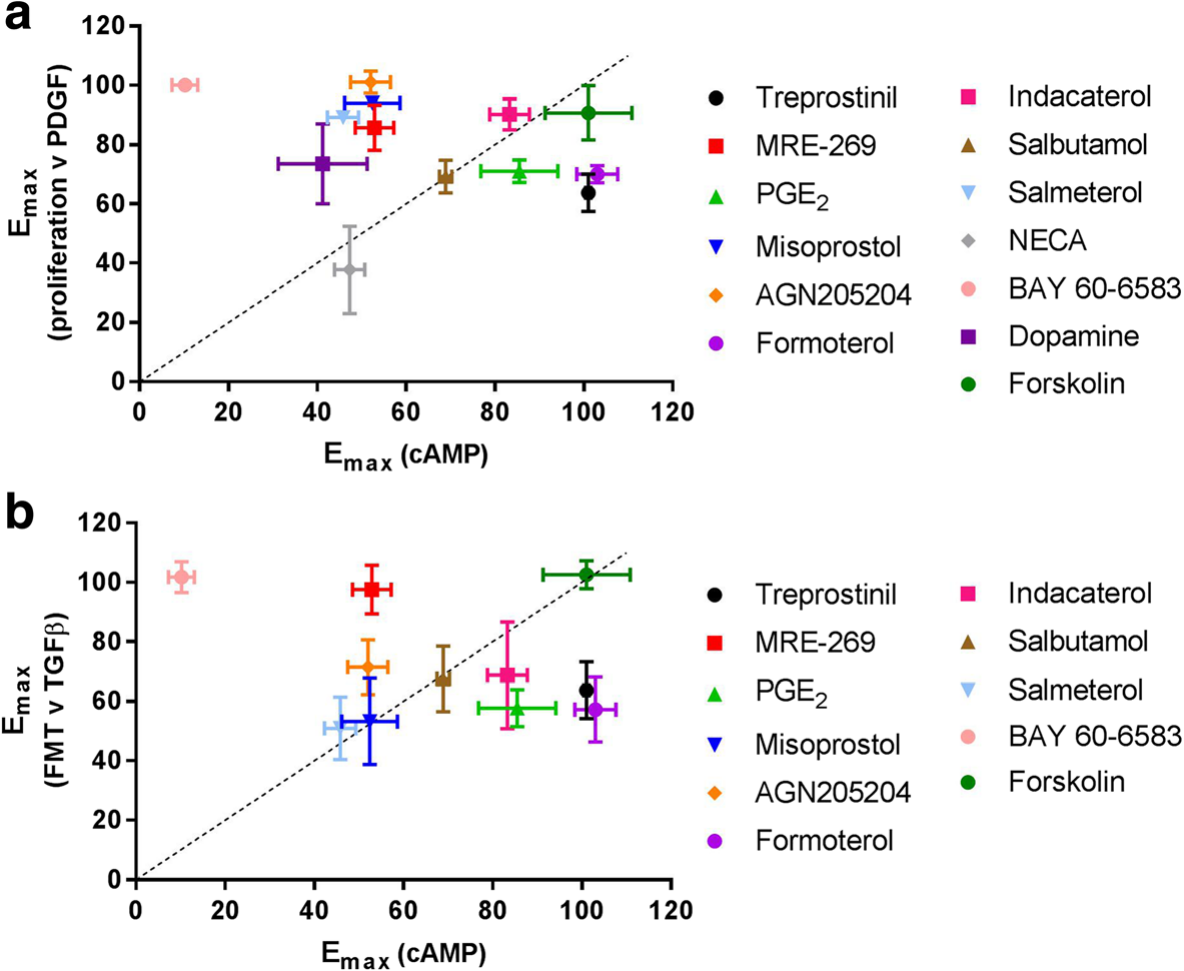
Correlation plot. Correlation between global cAMP accumulation and maximal inhibition of PDGF-mediated proliferation (**a**) or TGFβ-mediated FMT (**b**). Pearson correlation, dashed line = line of unity

Because the assays like the simple, homogenous second messenger system used here are often utilised in high throughput screening to identify novel agonists for cAMP accumulation, we wanted to explore the relevance of this method for predicting the inhibition of HLF proliferation and differentiation. To do this, we generated correlation plots for maximal levels of cAMP generation against the maximal inhibition of proliferation or FMT (Fig. 7). There was no significant correlation between the amount of cAMP accumulation and the degree of inhibition of PDGF-mediated proliferation (r^2^ = 0.06, *p* = 0.41), or TGFβ-mediated FMT (r^2^ = 0.06, *p* = 0.46*)*. This was most apparent with formoterol and treprostinil, which despite generating high levels of cAMP were relatively ineffective at inhibiting proliferation and differentiation. In contrast, BAY60–6583 and MRE-269 exhibited higher efficacy for the inhibition of proliferation and FMT than cAMP accumulation.

## Discussion

IPF is a devastating disease, and with current therapeutics only able to slow disease progression and being associated with severe side effects, further research in IPF aetiology and drug discovery is urgently required. A key feature of IPF is airway remodelling which is driven by fibroblast proliferation and differentiation. This leads to the formation of fibroblast and myofibroblast foci that excrete excessive amounts of extracellular matrix resulting in airway stiffening and loss of pulmonary function. It is this airway remodelling that is associated with disease severity [2, 22] suggesting that targeting the inhibition of key fibroblast activities may lead to novel therapeutic approaches for IPF. There is an evolving body of evidence demonstrating cAMP as a potential target for the treatment of IPF, however it is unclear how acute cAMP accumulation is able to affect the chronic phenotypic responses involved in fibrosis, such as cell proliferation and differentiation.

Approved therapies and novel approaches undergoing clinical trials in IPF take the approach of inhibiting a range of pro-fibrotic stimuli, often GF-RTK combinations that predominantly signal via the MAPK pathway. Using fibroblast proliferation to model aspects of lung fibrosis, we demonstrated that inhibition of the MAPK pathway completed blocked PDGF-mediated proliferation, and that ERK phosphorylation could be completely inhibited by increasing global cAMP. For this reason, we wanted to explore the expression of G_s_-coupled GPCR in HLF, as well as their ability to increase cAMP and subsequently inhibit HLF proliferation and differentiation in order to identify new targets to treat IPF that will complement existing therapies.

We observed mRNA expression of a number of different G_s_-coupled GPCR in fibroblasts, including related receptor families such as those for prostaglandins (IP, EP_2_, EP_4_) and adenosine (A_2A_ and A_2B_), as well as the MC1, β_2_ adrenoceptor and dopamine-1 receptors. Importantly, not all of these receptors resulted in functional cAMP responses, as measured by high throughput, homogenous assay systems. This highlights the need to look not only at mRNA levels, but protein expression and function as well to confirm active receptors are present.

To model chronic aspects of IPF we chose to study HLF proliferation and differentiation, as HLF are key mediators of fibrosis and contain human receptors and signalling proteins at relevant levels. Proliferation was monitored using two complimentary methods, BrdU incorporation into newly synthesized DNA of actively proliferating cells, and direct cell counting. Differentiation was monitored using an FMT assay that measures the increased expression of αSMA that indicates a myofibroblast phenotype. Using the BrdU incorporation assay we were able to quantify proliferation in response to both PDGF and serum (which contains multiple growth factors and proliferative stimuli) at an earlier stage compared to the cell counting method. This is likely due to the different sensitivities of the assays as well as the time course of responses being measured. We also observed that full concentration-response curves for the inhibition of cell counting could not be obtained for all ligands. For this reason, we focused on the BrdU incorporation and FMT assays to explore the responses further.

Using these models, we have demonstrated that selected agonists for the IP, β_2_, EP_2_, EP_4_, and A_2B_ receptors, as well as forskolin, an activator of AC, were able to robustly inhibit HLF proliferation and differentiation. Interestingly, some agonists such as MRE-269, AGN205204 and BAY 60–6583 were able to fully inhibit either proliferation or FMT, despite being partial agonists for cAMP accumulation. This demonstrates that many of the agonists we have tested in this study have the potential to inhibit multiple processes that contribute to IPF clinically. It is important to note however that these studies were performed on fibroblasts from healthy donors, and therefore these results should be confirmed in fibroblasts from IPF patients, which may differ in their established phenotype.

We were able to demonstrate using bother fibroblast proliferation and differentiation that cAMP accumulation does not correlate with the degree of inhibition of chronic phenotypic responses. This highlights the importance of developing novel treatments for IPF on the basis of their chronic effects rather than acute cAMP responses. For example, although iloprost has been described as a potential treatment for IPF due to its protective effects against bleomycin-induced pulmonary fibrosis in mice [23], our data suggests this may not be the optimal IP agonist for the treatment of IPF.

A number of the GPCR we identified here have previously been implicated in the treatment of IPF or other chronic lung diseases. The endogenous EP receptor agonist PGE_2_ has been shown to inhibit cellular markers of fibrosis [14, 16], and its synthetic analogue 16, 16-dimethyl-PGE2 protects against bleomycin induced injury [24]. This coupled with diminished PGE_2_ signalling in lung fibrosis [25], and the strong inhibition of proliferation we observe with selective agonists suggests that EP_2_ &/or EP_4_ agonists are strong candidates for novel IPF treatments. Recent studies have also identified the A_2B_ receptor as an important target in the regulation of both acute and chronic lung disease, with agonists being considered for the treatment of acute lung injury and antagonists being considered for the treatment of chronic lung diseases [26–28]. In agreement with these findings, we observed that the selective A_2B_ agonist BAY 60–6583, although not very potent, showed robust inhibition of HLF proliferation.

Currently, inhaled β_2_ adrenoceptor agonists are widely used for the treatment of lung diseases such as chronic obstructive pulmonary disease (COPD), providing relief by inducing bronchodilation of airway smooth muscle. We have shown here that the long acting β_2_ adrenoceptor agonists’ salmeterol and indacaterol may also act as anti-fibrotic agents in IPF through the robust inhibition of HLF proliferation and differentiation.

Although we did not observe any acute cAMP accumulation with the PDE inhibitor IBMX, we did observe some inhibition of both proliferation and FMT at higher concentrations. In the isolated fibroblasts there may not be sufficient basal cAMP turnover in order for PDE inhibitors to substantially increase levels of cAMP during the shorter cAMP assay, but with the extended incubation for the phenotypic assays (24 or 48 h), IBMX may be able to cause some accumulation of cAMP that is driving this inhibitory effect. In support of this, PDE inhibitor use is being explored for IPF and they have been shown to be effective in the bleomycin model [18, 19]. It would therefore be interesting to determine whether co-administration with GPCR agonists could further boost the anti-proliferative capacity of the agonists tested here.

The mechanisms behind the apparent disconnect we observed between acute cAMP accumulation and inhibition of chronic phenotypic responses is not yet clear, however there are a number of factors that need to be considered. Firstly, proliferation and differentiation were monitored over a longer time period than the cAMP accumulation assays, so it is possible that the agonists tested were degrading over time leading to loss of response. We have previously demonstrated that for treprostinil and MRE-269 > 90% of the original concentration of agonist remains after 24 h incubation with HLF (unpublished data), suggesting that it is unlikely that ligand degradation is contributing to the effect we have seen.

Another aspect of GPCR signalling that may be contributing to the responses we have observed is the ability to signal through multiple G proteins or effector molecules. It has now been well characterized that β-arrestin, once recruited to a GPCR, may initiate a cascade of G protein-independent signalling [29], the best characterized of which is the MAPK/ERK cascade. In these cells activation of cAMP signalling leads to inhibition of proliferation, most likely via inhibition of the ERK phosphorylation. This suggests that this promiscuous signalling is unlikely to be contributing to the disconnect we observed between cAMP and inhibition of proliferation, although it should be explored further. Forskolin was able to robustly inhibit both fibroblast proliferation and differentiation, further supporting a direct role for the cAMP pathway in the inhibition of these chronic phenotypic responses. It may be that the spatial or temporal control of these cAMP signals is more important in regulating downstream, chronic effects rather than the absolute levels of cAMP generated.

The temporal characteristics of the cAMP response can be controlled directly through the kinetics or duration of the cAMP response, or through time-dependent internalization and desensitization of activated receptors. Agonists such as treprostinil and iloprost which are full agonists in the cAMP accumulation assay may undergo a higher degree of desensitization than the partial agonist MRE-269. This could explain why there is a reversal of intrinsic activity for these ligands in the two assays. However, recent evidence from chronic cAMP studies suggests that regardless of their efficacy, all agonists will produce the same relative degree of functional desensitization [30]. In support of this, indacaterol, which elicits 80% maximal response in cAMP is as effective at inhibiting proliferation as the lower efficacy agonist salmeterol, which elicits a 40% maximal cAMP response. Interestingly, both these compounds are long-acting β_2_ adrenoceptor agonists, which suggests a role for signalling kinetics in mediating chronic phenotypic events. This has been demonstrated previously for the regulation of gene transcription after GPCR activation, where the kinetics or duration of second messenger responses was shown to drive transcriptional activity [31, 32].

Compartmentalized cAMP signalling may also contribute to receptor/ligand-dependent downstream effects. Selective activation of specific intracellular pools of cAMP can occur through physical intracellular barriers, such as the A-kinase-anchoring proteins (AKAP) [33–35], or through persistent signalling of agonist-receptor complexes after internalization. This persistent signalling has been described for the G_i_-coupled S1P_1_ receptor [36] and more recently with the β_2_ adrenoceptor [37]. Further studies are required to determine if the duration and location of the cAMP signalling, rather than the magnitude, is important for long-term phenotypic responses.

## Conclusion

In conclusion, in these studies we have demonstrated that selected agonists at the IP, β_2_, EP_2_, EP_4_, and A_2B_ receptors have the potential to inhibit pro-fibrotic processes such as fibroblast proliferation and differentiation, implying a potential for disease modifying effects in IPF. From these results it appears that although the cAMP response is important in driving the anti-fibrotic effects we have observed, there appears to be a threshold of cAMP that is required to inhibit proliferation and differentiation over which any further increases of cAMP do not drive further inhibition. For this reason, the magnitude of acute, global cAMP accumulation does not accurately predict the degree of inhibition of proliferation or differentiation suggesting additional factors, such as the spatio-temporal control of cAMP are important. Further research is required to understand this disconnect, which will aid in the discovery of novel, therapeutically beneficial treatments for IPF.

## Additional file

Additional file 1: Additional data describing full GPCR expression analysis in human lung fibroblasts and chemical structures of ligands used in these studies. Further characterization of these ligands in an imaging-based nuclear count assay and cell viability assay is presented. (DOCX 1357 kb)

## Abbreviations

A2A: Adenosine 2A
A2B: Adenosine 2B
AC: Adenylyl cyclase
AKAP: A-kinase anchoring protein
cAMP: 3′, 5′ cyclic adenosine monophosphate
COPD: Chronic obstructive pulmonary disease
CT: Cycle threshold
DMEM: Dulbecco’s modified eagle medium
dPBS: Dulbecco’s phosphate buffered saline solution
ECM: Extracellular matrix
ERK: Extracellular signal–regulated kinase
FBS: Foetal bovine serum
FMT: Fibroblast to myofibroblast transdifferentiation
FRET: Fluorescence resonance energy transfer
GF: Growth factor
GPCR: G protein-coupled receptors
HBSS: Hank’s Buffered Salt Solution
HLF: Human lung fibroblasts
HSA: Human serum albumin
IP: Prostacyclin
IPF: idiopathic pulmonary fibrosis
LDH: lactose dehydrogenase
MAPK: Mitogen-activated protein kinase
MC1: Melanocortin-1
PAH: Pulmonary arterial hypertension
PDE: Phosphodiesterase
PDGF: Platelet-derived growth factor
PFA: Paraformaldehyde
PGE_2_: Prostaglandin E_2_
PKA: Protein kinase A
RTK: Receptor tyrosine kinase
SEM: Standard error of mean
TGFβ: Transforming growth factor β
VEGF: Vascular endothelial growth factor
α-MSH: Alpha melanocortin stimulating hormone

## References

[1] Navaratnam V, Fleming KM, West J, Smith CJ, Jenkins RG, Fogarty A, Hubbard RB (2011). The rising incidence of idiopathic pulmonary fibrosis in the U.K. Thorax.

[2] Katzenstein AL, Myers JL. Idiopathic pulmonary fibrosis: clinical relevance of pathologic classification. Am J Respir Crit Care Med. 1998;157 10.1164/ajrccm.157.4.9707039 9563754

[3] Wolters PJ, Collard HR, Jones KD (2014). Pathogenesis of idiopathic pulmonary fibrosis. Annu Rev Pathol.

[4] Chaudhary NI, Roth GJ, Hilberg F, Müller-Quernheim J, Prasse A, Zissel G, Schnapp A, Park JE (2007). Inhibition of PDGF, VEGF and FGF signalling attenuates fibrosis. Eur Respir J.

[5] Iyer SN, Gurujeyalakshmi G, Giri SN (1999). Effects of Pirfenidone on transforming growth factor-β gene expression at the transcriptional level in bleomycin hamster model of lung fibrosis. J Pharmacol Exp Ther.

[6] Iyer SN, Hyde DM, Giri SN (2000). Anti-inflammatory effect of Pirfenidone in the bleomycin-hamster model of lung inflammation. Inflammation.

[7] Noble PW, Albera C, Bradford WZ, Costabel U, Glassberg MK, Kardatzke D, King TE, Lancaster L, Sahn SA, Szwarcberg J (2011). Pirfenidone in patients with idiopathic pulmonary fibrosis (CAPACITY): two randomised trials. Lancet.

[8] Richeldi L, Costabel U, Selman M, Kim DS, Hansell DM, Nicholson AG, Brown KK, Flaherty KR, Noble PW, Raghu G (2011). Efficacy of a tyrosine kinase inhibitor in idiopathic pulmonary fibrosis. N Engl J Med.

[9] King TE, Bradford WZ, Castro-Bernardini S, Fagan EA, Glaspole I, Glassberg MK, Gorina E, Hopkins PM, Kardatzke D, Lancaster L (2014). A phase 3 trial of pirfenidone in patients with idiopathic pulmonary fibrosis. N Engl J Med.

[10] Richeldi L, du Bois RM, Raghu G, Azuma A, Brown KK, Costabel U, Cottin V, Flaherty KR, Hansell DM, Inoue Y (2014). Efficacy and safety of nintedanib in idiopathic pulmonary fibrosis. N Engl J Med.

[11] Sun Y, Liu WZ, Liu T, Feng X, Yang N, Zhou HF (2015). Signaling pathway of MAPK/ERK in cell proliferation, differentiation, migration, senescence and apoptosis. J Recept Signal Transduct Res.

[12] Nikam VS, Wecker G, Schermuly R, Rapp U, Szelepusa K, Seeger W, Voswinckel R: Treprostinil Inhibits Adhesion and Differentiation of Fibrocytes via cAMP and Rap Dependent ERK Inactivation. *Am J Res Cell Mol Biol* 2011:2010-0240OC. 10.1165/rcmb.2010-0240OC 21278326

[13] Stork PJ, Schmitt JM (2002). Crosstalk between cAMP and MAP kinase signaling in the regulation of cell proliferation. Trends Cell Biol.

[14] Fine A, Goldstein RH (1987). The effect of PGE2 on the activation of quiescent lung fibroblasts. Prostaglandins.

[15] Kohyama T, Ertl RF, Valenti V, Spurzem J, Kawamoto M, Nakamura Y, Veys T, Allegra L, Romberger D, Rennard SI (2001). Prostaglandin E(2) inhibits fibroblast chemotaxis. Am J Physiol Lung Cell Mol Physiol.

[16] Kolodsick JE, Peters-Golden M, Larios J, Toews GB, Thannickal VJ, Moore BB (2003). Prostaglandin E2 inhibits fibroblast to myofibroblast transition via E. Prostanoid receptor 2 signaling and cyclic adenosine monophosphate elevation. AmJRespirCell MolBiol.

[17] Liu X, Ostrom RS, Insel PA. cAMP-elevating agents and adenylyl cyclase overexpression promote an antifibrotic phenotype in pulmonary fibroblasts. *AmJPhysiol* Cell Physiol. 2004;286:C1089–99. 10.1152/ajpcell.00461.2003 15075208

[18] Cortijo J, Iranzo A, Milara X, Mata M, Cerda-Nicolas M, Ruiz-Sauri A, Tenor H, Hatzelmann A, Morcillo EJ (2009). Roflumilast, a phosphodiesterase 4 inhibitor, alleviates bleomycin-induced lung injury. Br J Pharmacol.

[19] Udalov S, Dumitrascu R, Pullamsetti SS, Al-tamari HM, Weissmann N, Ghofrani HA, Guenther A, Voswinckel R, Seeger W, Grimminger F, Schermuly RT (2010). Effects of phosphodiesterase 4 inhibition on bleomycin-induced pulmonary fibrosis in mice. BMC Pulm Med.

[20] Groot-Kormelink PJ, Fawcett L, Wright PD, Gosling M, Kent TC (2012). Quantitative GPCR and ion channel transcriptomics in primary alveolar macrophages and macrophage surrogates. BMCImmunol.

[21] Alexander SP, Mathie A, Peters JA (2011). Guide to receptors and channels (GRAC), 5th edition. Br J Pharmacol.

[22] Talmadge EKJ, Schwarz MI, Brown K, Tooze JA, Colby TV, James AWJ, Flint A, Thurlbeck W, Cherniack RM (2001). Idiopathic pulmonary fibrosis. Am J Respir Crit Care Med.

[23] Zhu Y, Liu Y, Zhou W, Xiang R, Jiang L, Huang K, Xiao Y, Guo Z, Gao J (2010). A prostacyclin analogue, iloprost, protects from bleomycin-induced pulmonary fibrosis in mice. RespirRes.

[24] Failla M, Genovese T, Mazzon E, Fruciano M, Fagone E, Gili E, Barera A, La RC, Conte E, Crimi N (2009). 16,16-dimethyl prostaglandin E2 efficacy on prevention and protection from bleomycin-induced lung injury and fibrosis. AmJRespirCell MolBiol.

[25] Wilborn J, Crofford LJ, Burdick MD, Kunkel SL, Strieter RM, Peters-Golden M (1995). Cultured lung fibroblasts isolated from patients with idiopathic pulmonary fibrosis have a diminished capacity to synthesize prostaglandin E2 and to express cyclooxygenase-2. J Clin Invest.

[26] Eckle T, Faigle M, Grenz A, Laucher S, Thompson LF, Eltzschig HK (2008). A2B adenosine receptor dampens hypoxia-induced vascular leak. Blood.

[27] Eckle T, Grenz A, Laucher S, Eltzschig HK (2008). A2B adenosine receptor signaling attenuates acute lung injury by enhancing alveolar fluid clearance in mice. J Clin Invest.

[28] Sun CX, Zhong H, Mohsenin A, Morschl E, Chunn JL, Molina JG, Belardinelli L, Zeng D, Blackburn MR (2006). Role of A2B adenosine receptor signaling in adenosine-dependent pulmonary inflammation and injury. J Clin Invest.

[29] Lefkowitz RJ, Shenoy SK (2005). Transduction of receptor signals by {beta}-Arrestins. Science.

[30] Rosethorne EM, Bradley ME, Kent TC, Charlton SJ (2015). Functional desensitization of the beta 2 adrenoceptor is not dependent on agonist efficacy. Pharmacol Res Perspect.

[31] Baker JG, Hall IP, Hill SJ (2004). Temporal characteristics of cAMP response element-mediated gene transcription: requirement for sustained cAMP production. Mol Pharmacol.

[32] Rosethorne EM, Nahorski SR, Challiss RA (2008). Regulation of cyclic AMP response-element binding-protein (CREB) by Gq/11-protein-coupled receptors in human SH-SY5Y neuroblastoma cells. Biochem Pharmacol.

[33] Dema A, Perets E, Schulz MS, Deak VA, Klussmann E (2015). Pharmacological targeting of AKAP-directed compartmentalized cAMP signalling. Cell Signal.

[34] Torres-Quesada O, Mayrhofer JE, Stefan E (2017). The many faces of compartmentalized PKA signalosomes. Cell Signal.

[35] Langeberg LK, Scott JD (2015). Signalling scaffolds and local organization of cellular behaviour. Nat Rev Mol Cell Biol.

[36] Mullershausen F, Zecri F, Cetin C, Billich A, Guerini D, Seuwen K (2009). Persistent signaling induced by FTY720-phosphate is mediated by internalized S1P1 receptors. Nat Chem Biol.

[37] Irannejad R, Tomshine JC, Tomshine JR, Chevalier M, Mahoney JP, Steyaert J, Rasmussen SGF, Sunahara RK, El-Samad H, Huang B, von Zastrow M (2013). Conformational biosensors reveal GPCR signalling from endosomes. Nature.

