# Supplementary material for: The inhibition of human lung fibroblast proliferation and differentiation by Gs-coupled receptors is not predicted by the magnitude of cAMP response
Source: Respir Res. 2018 Apr 7;19:56. doi: 10.1186/s12931-018-0759-2 (PMC5889558; doi:10.1186/s12931-018-0759-2)
Supplement: Supplementary file 1 — Additional data describing full GPCR expression analysis in human lung fibroblasts and chemical structures of ligands used in these studies. Further characterization of these ligands in an imaging-based nuclear count assay and cell viability assay is presented. (DOCX 1357 kb) [file 12931_2018_759_MOESM1_ESM.docx]

**Supplementary Methods**

**Proliferation**

Proliferation of HLF was measured by nuclei count. Human lung fibroblasts (HLF) were seeded overnight at 4,000 cells/well in 96-well black ViewPlates, before being starved for 24 h in culture medium devoid of FBS. Proliferation was measured after incubation with a range of concentrations of FBS or PDGF, in DMEM supplemented with 0.1 % w/v HSA, for 24, 48, 72, or 96 h for nuclei count assay.

For anti-remodelling assays, cells were incubated for 48 h with a range of concentrations of test compounds in the presence of an EC_80_ concentration of FBS (1.8 % v/v). Following stimulation, cells were fixed with 4 % PFA and nuclei stained with Hoechst 33342 (1 μM) for 15 minutes at room temperature. Cells were then washed three times with dPBS before the plate was read using the widefield ImageXpress Micro microscope, with a Plan Fluor 4X objective, DAPI filter cube (excitation 400-418nm, emission 435-470nm), and 50 ms exposure time. Images were analysed using a nuclear count algorithm within the MetaXpress 5.3 software (Molecular Devices, California, USA). To account for the inter-assay variation in levels of proliferation in each experiment, data were normalized to the EC_80_ FBS response.

**Cell Viability Assay**

Cell viability was measured using caspase-3/7 expression as a marker of apoptosis after treatment for 24 h with test compounds. HLF were seeded overnight at 6,000 cells/well in 96-well black ViewPlates, before being starved for 24 hours in culture medium devoid of FBS. Cells were incubated for 24 hours in the presence of test compounds at the top concentrations used in the proliferation assays, or in the presence of 100 nM staurosporine to induce apoptosis.

The presence of the apoptotic marker caspase-3/7 was detected by incubating cells with 8 μM CellEvent™ Caspase-3/7 Red Detection Reagent in dPBS with 5 % FBS, for 30 minutes at 37°C. Following incubation, cells were fixed with 4 % paraformaldehyde and the nuclei stained with Hoechst 33342 (1 μM) for 15 minutes at room temperature. Cells were then washed three times with dPBS before the plate was read using the widefield ImageXpress Micro microscope, with a Plan Fluor 4X objective, DAPI filter cube (excitation 400-418nm, emission 435-470nm) with 50 ms exposure time, and Cy5 filter cube (excitation 604-644nm, emission 672-712nm) with 1000 ms exposure time. Images were analyzed using a multi wavelength cell scoring algorithm within the MetaXpress 5.3 software, which measures cell number using stained nuclei, and percent of these cells positive for caspase-3/7 from Cy5 readouts. To account for the inter-assay variation in levels of caspase 3/7 detected in each experiment, data were normalized to the staurosporine response.

Statistical significance across treatment groups was determined by using the one-way ANOVA with Dunnett’s multiple-comparison post hoc test.

**Supplementary Results**

**Table S1 mRNA expression of G protein-coupled receptors in human lung fibroblasts**

| **Receptor** | **Expression** | **Putative G Protein** | **Receptor** | **Expression** | **Putative G Protein** | **Receptor** | **Expression** | **Putative G Protein** |
| --- | --- | --- | --- | --- | --- | --- | --- | --- |
| Prostacyclin receptor | 0.053 ± 0.016 | Gs | Adenosine 1 | 0.012 ± 0.008 | Gi | Purinergic Receptor P2Y11 | 0.010 ± 0.003 | Gq |
| Prostaglandin E receptor 2 | 0.029 ± 0.001 | Gs | Complement component 5a receptor 1 | 0.004 ± 0.003 | Gi | Thromboxane A2 receptor | 0.009 ± 0.003 | Gq |
| Melanocortin 1 receptor | 0.012 ± 0.002 | Gs | Lysophosphatidic acid receptor 2 | 0.004 ± 0.002 | Gi | Prostaglandin E receptor 1 | 0.008 ± 0.004 | Gq |
| β_2_ adrenoceptor | 0.008 ± 0.004 | Gs | Chemokine (C-C motif) receptor 9 | 0.003 ± 0.006 | Gi | Endothelin receptor type A | 0.008 ± 0.002 | Gq |
| Adenosine 2B | 0.007 ± 0.003 | Gs | Opiate receptor-like 1 | 0.002 ± 0.001 | Gi | α_1A_-adrenergic | 0.003 ± 0.002 | Gq |
| Prostaglandin E receptor 4 | 0.004 ± 0.002 | Gs | Serotonin receptor 2C | 0.002 ± 0.002 | Gi | Coagulation Factor II Thrombin Receptor | 0.641 ± 0.213 | Gi+Gq |
| Dopamine receptor D1 | 0.004 ± 0.001 | Gs | GPR21 | 0.002 ± 0.000 | Gi | Coagulation factor II (thrombin) receptor-like 1 | 0.060 ± 0.040 | Gi+Gq |
| Adenosine 2A | 0.002 ± 0.001 | Gs | G protein-coupled estrogen receptor 1 | 0.002 ± 0.001 | Gi | Oxytocin receptor | 0.049 ± 0.035 | Gi+Gq |
| Cholinergic Receptor Muscarinic 2 | 0.196 ± 0.042 | Gi | Gastrin-releasing peptide receptor | 0.002 ± 0.001 | Gi | Melanin-concentrating hormone receptor 1 | 0.004 ± 0.001 | Gi+Gq |
| Sphingosine-1-phosphate receptor 2 | 0.101 ± 0.012 | Gi | Bradykinin receptor B1 | 0.135 ± 0.125 | Gq | Leukotriene B4 receptor 2 | 0.003 ± 0.000 | Gi+Gq |
| **Receptor** | **Expression** | **Putative G Protein** | **Receptor** | **Expression** | **Putative G Protein** | **Receptor** | **Expression** | **Putative G Protein** |
| GABA B receptor 1 | 0.070 ± 0.012 | Gi | Bradykinin receptor B2 | 0.135 ± 0.074 | Gq | Lysophosphatidic acid receptor 3 | 0.002 ± 0.002 | Gi+Gq |
| Purinergic Receptor P2Y13 | 0.048 ± 0.094 | Gi | Prostaglandin F receptor | 0.029 ± 0.008 | Gq | Leukotriene B4 receptor | 0.002 ± 0.001 | Gi+Gq |
| Sphingosine-1-phosphate receptor 1 | 0.023 ± 0.014 | Gi | Purinergic Receptor P2Y1 | 0.021 ± 0.016 | Gq | Lysophosphatidic acid receptor 6 | 0.005 ± 0.002 | G12 |
| Somatostatin receptor 1 | 0.012 ± 0.006 | Gi | Serotonin receptor 2B | 0.020 ± 0.013 | Gq | Lysophosphatidic acid receptor 1 | 0.304 ± 0.094 | Gi+Gq+G12 |
| Sphingosine-1-phosphate receptor 3 | 0.185 ± 0.043 | Gi+Gq+G13 | SUMO1/sentrin/SMT3 specific peptidase 3 | 0.073 ± 0.007 | ND | GPR161 | 0.019 ± 0.001 | ND |
| Frizzled homolog 7 | 0.218 ± 0.049 | ND | Histone deacetylase 3 | 0.065 ± 0.003 | ND | GPR125 | 0.018 ± 0.004 | ND |
| RARS - arginyl-tRNA synthetase | 0.212 ± 0.007 | ND | MAS Related GPR Family Member F | 0.064 ± 0.017 | ND | GPR173 | 0.014 ± 0.003 | ND |
| GPR124 | 0.206 ± 0.023 | ND | GPR137 | 0.062 ± 0.013 | ND | LanC lantibiotic synthetase component C-like 2 | 0.014 ±0.002 | ND |
| Frizzled homolog 6 | 0.201 ± 0.068 | ND | GPR153 | 0.061 ± 0.018 | ND | GPR1 | 0.013 ± 0.003 | ND |
| GPR176 | 0.169 ± 0.023 | ND | CD97 | 0.059 ± 0.007 | ND | Vomeronasal 1 receptor 1 | 0.012 ± 0.009 | ND |
| Coagulation factor II (thrombin) receptor-like 2 | 0.153 ± 0.074 | ND | Frizzled homolog 1 | 0.055 ± 0.022 | ND | GPRC5B | 0.011 ± 0.003 | ND |
| LONPL | 0.107 ± 0.017 | ND | GPRC5A | 0.046 ± 0.040 | ND | GPR135 | 0.010 ± 0.005 | ND |
| GPR126 | 0.102 ± 0.063 | ND | Frizzled homolog 4 | 0.044 ± 0.020 | ND | Leucine-Rich Repeat Containing G Protein-Coupled Receptor 4 | 0.008 ± 0.002 | ND |
| **Receptor** | **Expression** | **Putative G Protein** | **Receptor** | **Expression** | **Putative G Protein** | **Receptor** | **Expression** | **Putative G Protein** |
| Phosphoglycerate dehydrogenase | 0.098 ± 0.045 | ND | Frizzled homolog 2 | 0.029 ± 0.003 | ND | GPR68 | 0.007 ± 0.003 | ND |
| GPR37 | 0.087 ± 0.037 | ND | Adhesion G Protein-Coupled Receptor L4 | 0.028 ± 0.015 | ND | GPR56 | 0.007 ± 0.004 | ND |
| Latrophilin 2 | 0.083 ± 0.023 | ND | Smoothened homolog | 0.025 ± 0.006 | ND | GPR85 | 0.006 ± 0.001 | ND |
| LanC lantibiotic synthetase component C-like 1 | 0.081 ±0.006 | ND | Adhesion G Protein-Coupled Receptor B2 | 0.025 ± 0.008 | ND | Frizzled homolog 8 | 0.005 ± 0.002 | ND |
| Mitochondrial ribosomal protein L49 | 0.080 ± 0.016 | ND | Opsin 3 | 0.022 ± 0.010 | ND | Chemokine (C-C motif) receptor-like 1 | 0.005 ± 0.001 | ND |
| Frizzled homolog 5 | 0.004 ± 0.001 | ND | Latrophilin 1 | 0.003 ± 0.002 | ND | Calcitonin receptor-like | 0.002 ± 0.001 | ND |
| Cadherin EGF LAG Seven-Pass G-Type Receptor 1 | 0.004 ± 0.003 | ND | GPR146 | 0.002 ± 0.001 | ND | Cadherin EGF LAG Seven-Pass G-Type Receptor 3 | 0.002 ± 0.000 | ND |
| Olfactory receptor 2A4 | 0.003 ± 0.002 | ND |  |  |  |  |  |  |

**Table S1 Expression of endogenously expressed GPCR in HLF.** High density 384-well GPCR TaqMan arrays were run on HLF to monitor the expression of GPCRs. 8 housekeeping genes were included as controls in the arrays, 18S, ACTB, B2M, GAPDH, GUSB, HMBS, HPRT1, IPO8, PGK1, POLR2A, PPIA, RPLPO, TBP, AND TFRC. Expression is reported as 2 TO - ΔCT compared to the mean CT of the housekeeping genes and each value is the mean ± SD of 4 biological replicates.

ND – putative G protein coupling not yet defined

**Table S2 Cell Viability**

|  | % cells positive for caspase-3/7 | |
| --- | --- | --- |
|  | **Top conc** | **1:10 dilution** |
| Negative Control + 1% FBS | 4.27 ± 2.80 |  |
| Staurosporine (100 nM) | 54.8 ± 13.6 |  |
| Treprostinil (10 μM) | 0.76 ± 0.06 | 0.92 ± 0.25 |
| Iloprost (3 μM) | 0.88 ± 0.08 | 1.15 ± 0.29 |
| MRE-269 (10 μM) | 0.49 ± 0.21 | 0.49 ± 0.22 |
| PGE_2_ (10 μM) | 0.68 ± 0.29 | 0.53 ± 0.04 |
| Misoprostol (10 μM) | 0.54 ± 0.18 | 0.55 ± 0.04 |
| Butaprost (10 μM) | 0.80 ± 0.29 | 0.90 ± 0.39 |
| AGN-205204 (10 μM) | 0.57 ± 0.09 | 0.56 ± 0.09 |
| Formoterol (100 nM) | 0.67 ± 0.22 | 0.88 ± 0.48 |
| Indacaterol (1 μM) | 0.62 ± 0.01 | 0.62 ± 0.15 |
| Salbutamol (10 μM) | 0.46 ± 0.09 | 0.53 ± 0.12 |
| Salmeterol (100 nM) | 0.40 ± 0.12 | 0.66 ± 0.24 |
| NECA (30 μM) | 1.04 ± 0.20 | 0.67 ± 0.09 |
| BAY60-6583 (30 μM) | 0.62 ± 0.14 | 1.04 ± 0.38 |
| Dopamine (30 μM) | 0.83 ± 0.14 | 1.21 ± 0.34 |
| Forskolin (30 μM) | 1.26 ± 0.35 | 0.61 ± 0.15 |
| IBMX (10 μM) | 0.98 ± 0.27 | 1.24 ± 0.59 |

**Table S2 Cell Viability.** Measurement of cell viability using caspase-3/7 expression as a marker for apoptosis, after treatment with test compounds and staurosporine as a positive control. Data were normalized to the total number of cells in the field of view, and are expressed as means ± SEM for 3 independent experiments.

**Figure S1**


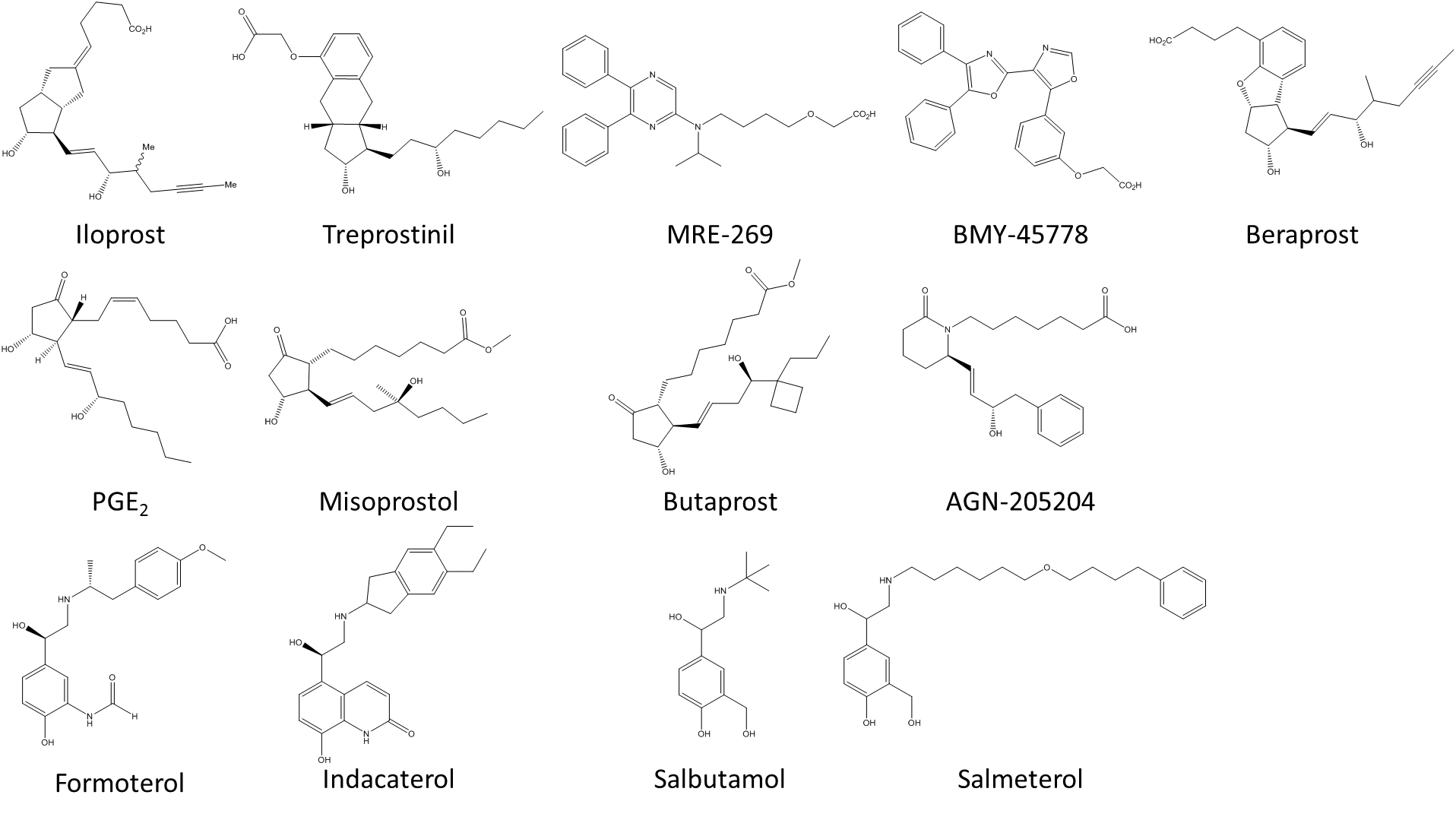


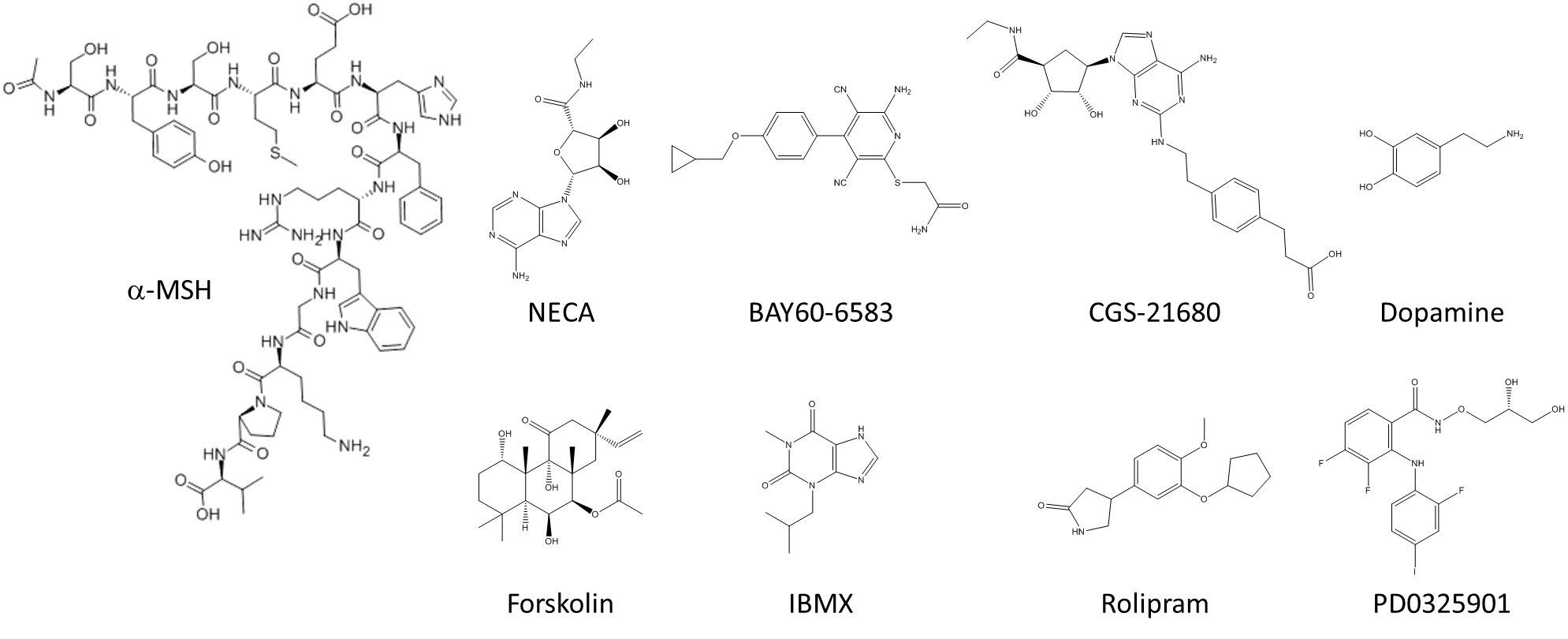


**Figure S1** Chemical structures of agonists used in this study

**Figure S2**

**Figure S2** fibroblast proliferation. Concentration-dependent increase in HLF proliferation following treatment with (A) serum or (B) PDGF for 24, 48, 72 or 96 h, measured using nuclei count assay. Data were normalized to maximal proliferation observed with 10 % serum at 96 h, and expressed as mean ± SEM for at least 3 independent experiments.

**Figure S3**

 **Figure S4** receptor-mediated inhibition of serum-mediated proliferation. Concentration effect curves for the inhibition of proliferation were determined in HLF following exposure to a range of agonists targeting the (A) IP and EP receptor, (B) other GPCR and (C) non-receptors, in the presence of an EC80 concentration of serum for 48 hours. For each individual experiment, data were normalized to the maximal proliferation observed with serum, and are expressed as means ± SEM for at least 3 independent experiments.
